# Synthetic Plantaricins Derived from *Lactiplantibacillus plantarum* KM2 Induce Cell Lysis of *Listeria monocytogenes*

**DOI:** 10.4014/jmb.2504.04006

**Published:** 2025-06-23

**Authors:** Seung-Eun Oh, Sojeong Heo, Minkyeong Kim, Yura Moon, Sumin Lee, Chaerin Park, Huieun Sung, Gawon Lee, Jina Kim, Moon-Hee Sung, Do-Won Jeong

**Affiliations:** 1 Department of Food and Nutrition, Dongduk Women’s University, Seoul 02748, Republic of Korea; 2 Insight View Tech, Hwasung 18469, Republic of Korea; 3 Kookmin Bio Corporation, Seoul 02826, Republic of Korea

**Keywords:** Plantaricin, *Listeria monocytogenes* ATCC 19111, antibacterial activity, growth inhibition, cell lysis

## Abstract

*Listeria monocytogenes* is a deadly foodborne pathogen that presents significant challenges in food safety management due to its high resilience in various environments. This study evaluated antibacterial activities of synthetic plantaricins derived from *Lactiplantibacillus plantarum* KM2 against *L. monocytogenes*. The minimum inhibitory concentrations of the five synthetic plantaricin combinations—spPlnA, spPlnJ, spPlnE&F, spPlnE&J, and spPlnJ&K—were 1.4, 1.5, 1.8, 1.6, and 1.6 μg/ml, respectively, Raman spectroscopy and transmission electron microscopy demonstrated that the synthetic plantaricins induced morphological alterations, including cell wall damage and cell lysis. Notably, spPlnE&F and spPlnE&J were shown to effectively disrupt the bacterial cell wall. Furthermore, assessments of antibacterial stability under varying temperatures and pH conditions showed that plantaricin combinations maintained their efficacy at pH levels ranging from 4 to 7 and temperatures below 40°C. These findings suggest that synthetic plantaricins have strong potential as natural preservatives in food applications, offering an effective approach to targeting specific pathogens and enhancing food safety.

## Introduction

*Listeria* (*L*.) *monocytogenes* is a serious foodborne pathogen responsible for listeriosis, a disease with a high mortality rate, particularly affecting the elderly, immunocompromised individuals, and pregnant women [1, 2]. Since its identification in the early 20th century, *L. monocytogenes* has posed significant public health risks, frequently contaminating food products such as dairy, meats, and fresh produce often due to inadequate handling and processing procedures [3, 4]. The pathogen’s resilience under various environmental conditions, including refrigeration temperatures and high salt concentrations, complicates control efforts during food processing [5]. Upon ingestion, *L. monocytogenes* can cross the intestinal barrier and disseminate throughout the host, leading to severe infections such as septicemia and meningitis [6, 7]. Therefore, effective intervention strategies are essential to prevent contamination and maintain food safety [8].

Bacteriocins have been studied as natural antimicrobial agents due to their ability to selectively inhibit pathogenic bacteria, offering a mode of action distinct from antibiotics [9]. Unlike antibiotics, which often lead to the development of resistance, bacteriocins possess the advantage of not inducing bacterial resistance [10]. Among them, nisin—a peptide-based bacteriocin approved for use in over 50 countries—is widely employed as a natural food preservative [11]. However, because nisin requires post-translational modifications for its bioactivity, it is typically harvested from its native producer, *Lactococcus lactis*. In contrast, bacteriocins that do not undergo post-translational modification can be produced via recombinant protein expression systems, enabling scalable production without dependence on specific producer strains. Bacteriocins produced by a range of bacteria, particularly lactic acid bacteria, have demonstrate potent antibacterial effects against Gram-positive organisms such as *L. monocytogenes* [12, 13]. Notable examples include nisin from *L. lactis* [14], Pediocin from *Pediococcus* species, mundticin from *Enterococcus mundtii* [15], and Bacillusin from *Bacillus velezensis* [16], all of which have been reported to inhibit the growth of *L. monocytogenes*. Bacteriocin-producing microorganisms can be utilized as starter cultures in fermented foods. Additionally, fractions containing these bacteriocins could be used as biopreservatives [17].

In a previous study, *Lactiplantibacillus* (*Lb.*) *plantarum* KM2 was isolated from dry-aged Korean beef [18]. Genome analysis confirmed the presence of a complete operon encoding the bacteriocin plantaricin [18, 19]. Antimicrobial activity tests against nine food spoilage and foodborne pathogens showed no inhibitory effect of *Lb. plantarum* KM2 on the growth of *L. monocytogenes* [18, 19]. However, when synthetic plantaricins derived from *Lb. plantarum* KM2 were tested using the disk diffusion method, growth inhibition of *L. monocytogenes* was observed [19]. Notably, plantaricin A, an autoinducer for plantaricin [20], exhibited higher activity than two-peptide bacteriocins plantaricin EF and JK [19]. Therefore, this study aimed to elucidate the inhibition mechanism of *L. monocytogenes* using synthetic plantaricin combinations, spPlnA, spPlnJ, spPlnE&F, spPlnE&J, and spPlnJ&K, that demonstrated strong antibacterial activity in a previous study.

## Materials and Methods

### Synthetic Plantaricins

In this study, five synthetic plantaricin combinations—spPlnA, spPlnJ, spPlnE&F, spPlnE&J, and spPlnJ&K—were employed to investigate their antibacterial mechanisms against *L. monocytogenes* [19]. These combinations consisted of plantaricin peptides (spPlnA, spPlnE, spPlnF, spPlnJ, and spPlnK) synthesized without signal peptides, based on amino acid sequences derived from genome of *Lb. plantarum* KM2 (GenBank Accession No. CP069282–CP069287; KCTC 14637BP), with a focus on mature peptide regions. Peptide synthesis was carried out using a Symphony X automated peptide synthesizer (Protein Technologies, Inc., USA), and the structural accuracy and molecular weights of the peptides were verified using Axima Assurance MALDI–TOF mass spectrometry (Shimadzu, Japan). To ensure consistency in experimental conditions, all peptide stock solutions were prepared in ddH_2_O following standardized protocols to reduce variability between samples.

### Antibacterial Bacterial Minimum Inhibitory Concentration

The minimum inhibitory concentration (MIC) against *L. monocytogenes* ATCC 19111 was determined by the broth microdilution method. Synthetic plantaricin samples–spPlnA (1.4 mg/ml), spPlnJ (1.8 mg/ml), spPlnE&F (1.6 mg/ml), spPlnE&J (1.6 mg/ml), spPlnJ&K (1.5 mg/ml)–were prepared as stock solutions as approximately 0.5 μM, adjusted based on their molecular weight. After preparing synthetic plantaricins by serial two-fold dilution, 20 μl was added to each well of a 96-microwell plate. *L. monocytogenes* ATCC 19111 was cultured in tryptic soy broth (TSB; Becton, Dickinson & Co., USA) until it reached an optical density at 600 nm (OD_600_) of 0.5. The culture was diluted 1:100 with fresh TSB. Then 180 μl of the diluted culture was added to each well of a 96-microwell plate. The MIC of the synthetic plantaricin was recorded as the lowest concentration at which no visible growth of *L. monocytogenes* ATCC 19111 was observed after incubation at 37°C for 24 h. MIC results were confirmed through at least three independent tests. Nisin (N5764, Sigma-Aldrich, USA) from *Lactococcus lactis* was used as a positive control. It was dissolved and diluted using dimethyl sulfoxide (D8418, Sigma-Aldrich). The negative control consisted of the same volume of ddH_2_O. All experiments were performed in triplicate using independent cultures.

### Raman Spectroscopy

*L. monocytogenes* ATCC 19111 was treated overnight with five synthetic plantaricin samples, 0.7 μg/ml spPlnA, 0.7 μg/ml spPlnJ, 0.9 μg/ml spPlnE&F, 0.8 μg/ml spPlnE&J, and 0.8 μg/ml spPlnJ&K—equivalent to half of their MIC values—and subsequently freeze-dried. Each sample was analyzed using Raman spectroscopy (RAMANtouch; Nanophoton Co., Japan) with a 532 nm single-mode diode laser. A 50× objective lens (50× magnification, numerical aperture 0.5, working distance 500 μm; Nikon, Japan) was used to focus the laser beam. Raman measurements were conducted at a laser power of 2.9 mW and an exposure time of 10 sec per sample. After spectral acquisition, multiple scatter correction was applied for baseline adjustment before performing principal component analysis (PCA). PCA models were constructed to differentiate and classify bacterial samples treated with different plantaricins based on their spectral features. Principal components identified directions of the greatest variation within the data, thereby revealing major distinctions among samples treated with different combinations of plantaricins. To validate results from PCA, partial least-squares discriminant analysis (PLS-DA) was performed as a supervised classification method, optimizing separation between treated and control samples by reducing dimensionality of the data and maximizing correlations between spectral variables and sample categories. All data processing, including baseline correction, PCA, and PLS-DA, was conducted using SIMCA-P software version 17.0 (Umetrics, Sweden). Raman peaks and antibacterial activity were subjected to analysis of variance to determine their statistical significance (*p* < 0.05). The statistical robustness of PCA and PLS-DA was verified by Hotelling T2 analysis.

### Transmission Electron Microscopy (TEM) Analysis

*L. monocytogenes* ATCC 19111 samples treated with synthetic plantaricins were fixed with 2.5% glutaraldehyde and 2% paraformaldehyde in a 0.1 M cacodylate buffer (pH 7.0) overnight at 4°C. Following fixation, samples were washed with a 0.05 M cacodylate buffer and then post-fixed with 1% osmium tetroxide at 4°C for 1.5 h, followed by immersion in 0.5% uranyl acetate for overnight staining. These fixed samples were dehydrated through a series of graded ethanol solutions (30%, 50%, 70%, 90%, and 100%) to ensure complete water removal essential to maintain structural integrity during the resin embedding process. Dehydrated samples were embedded in Spurr’s epoxy resin and polymerized at 70°C for 12 h. Resin blocks were sectioned to a thickness of 70 nm using an ultramicrotome (EM UC7; Leica Microsystems, Germany). Ultrathin sections were mounted onto 200-mesh copper grids and stained with uranyl acetate for 20 min, followed by lead citrate staining for 5 min. These stained sections were observed under a JEM-1010 TEM operating at 80 kV (Jeol, Japan), focusing on identifying structural alterations such as disruptions to the cell membrane and evidence of cell lysis.

### Effects of Temperature, pH, and Enzyme

To evaluate the stability of synthetic plantaricins under various temperature, pH, and enzymatic conditions, a disc diffusion assay was performed. *L. monocytogenes* ATCC 19111 was cultured until the OD_600_ reached 0.5. A 100 μl aliquot of the culture was then spread onto Tryptic Soy Agar (Becton, Dickinson, and Co.). A sterilized paper disc (8 mm, ADVANTEC, Japan) was placed on the agar surface and 20 μl of synthetic plantaricin pre-treated under specific temperature, pH, and enzyme conditions was applied to achieve a final concentration at half of the MIC (0.7 μg/ml for spPlnA, 0.7 μg/ml for spPlnJ, 0.9 μg/ml for spPlnE&F, 0.8 μg/ml for spPlnE&J, and 0.8 μg/ml for spPlnJ&K). To assess stability of temperature on bacteriocin activity, purified bacteriocins were incubated in a water bath at 20, 30, or 40 °C for 12 h. The antimicrobial activity of each temperature-treated sample was then evaluated using the disc diffusion assay. For pH stability testing, synthetic bacteriocins were adjusted to pH 4, 5, 6, or 7 using a Tris buffer solution (Genomic Base, Republic of Korea). These pH-adjusted solutions were filtered through 0.22-μm membranes and 20 μl of each was applied to a sterilized disc. To examine enzyme susceptibility, proteinase K, α-amylase, and lysozyme were prepared at a concentration of 20 mg/ml. Each enzyme was added to synthetic plantaricin at a concentration of 4 units/g, followed by incubation at 30°C for 12 h. Enzyme activity was then halted by heating samples at 100°C for 5 min, followed by rapid cooling on ice. Antimicrobial activity was determined by measuring the diameter of the clear zone surrounding each disc.

### Statistical Analysis

Duncan’s multiple range test following one-way analysis of variance (ANOVA) was used to evaluate significant differences between average values of stability of plantaricin under various treatments. Values with *p* < 0.05 were considered to be statistically significant. All analyses were carried out using the SPSS software version 27.0 (IBM SPSS Statistics, USA).

## Results

### MICs of Synthetic Plantaricins against *L. monocytogenes* ATCC 19111

The antibacterial effects of five synthetic plantaricins-spPlnA, spPlnE, spPlnF, spPlnJ, and spPlnK-were assessed individually and in combination against *L. monocytogenes* ATCC 19111 in a previous study [19]. It was confirmed that single combinations spPlnA and spPlnJ, as well as the dual combinations spPlnE&F, spPlnE&J, and spPlnJ&K, exhibited strong antimicrobial activities against *L. monocytogenes* ATCC 19111 [19]. Based on these results, MICs against *L. monocytogenes* ATCC 19111 of five bacteriocin combinations were determined. It was found that spPlnA exhibited a strong inhibitory activity with an MIC value of 1.4 μg/ml, indicating its high potency at a relatively lower concentration. MIC values of spPlnJ, spPlnE&F, spPlnE&J, and spPlnJ&K were 1.5 μg/ml, 1.8 μg/ml, 1.6 μg/ml, and 1.6 μg/ml, respectively. In comparison, the MIC value of nisin, used as a positive control, was confirmed to be 8.4 μg/ml. These results suggest that synthetic plantaricins exhibit higher antimicrobial activities against L. monocytogens than nisin.

### Analyzing Effects of Synthetic Plantaricins on *L. monocytogenes* ATCC 19111 Using Raman Spectroscopy

**Fig. 1:**
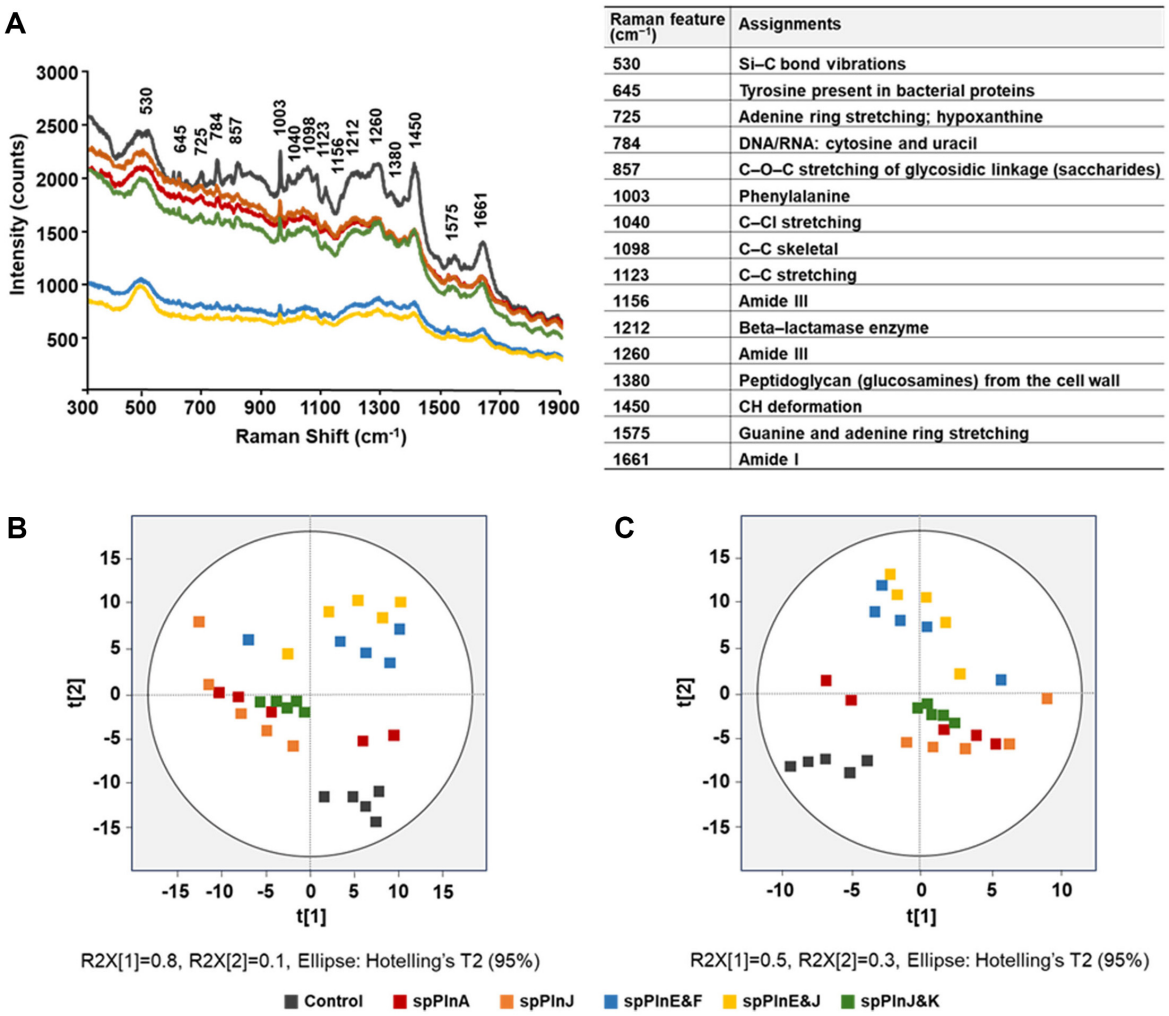
Raman spectra and multivariate analysis of *Listeria monocytogenes* treated with synthetic plantaricins. Raman spectra (**A**) principal component analysis (PCA; **B**), and partial least-squares discriminant analysis (PLS−DA; **C**) of *L. monocytogenes* ATCC 19111 treated with synthetic plantaricins are shown. Spectral data within the 300 to 1,900 cm^−1^ range were used for both PCA and PLS−DA.

Effects of five plantaricins, spPlnA, spPlnJ, spPlnE&F, spPlnE&J, and spPlnJ&K, against *L. monocytogenes* ATCC 19111 were determined using Raman spectroscopy. These synthetic plantaricins were used to treat *L. monocytogenes* ATCC 19111 at concentrations set to half of their respective MIC values: 0.7 μg/ml for spPlnA, 0.7 μg/ml for spPlnJ, 0.9 μg/ml for spPlnE&F, 0.8 μg/ml for spPlnE&J, and 0.8 μg/ml for spPlnJ&K. Raman spectra of *L. monocytogenes* treated with plantaricins showed changes in the detected peaks compared to untreated control (Fig. 1). Notable peaks were observed at 530, 645, 725, 784, 857, 1003, 1040, 1098, 1123, 1156, 1212, 1260, 1380, 1450, 1575, and 1661 cm^-1^ [21-29]. Those peaks corresponded to Si-C bond vibrations, Tyrosine present in bacterial proteins, Adenine ring stretching; hypoxanthine, DNA/RNA: cytosine and uracil, C–O–C stretching of glycosidic linkage (saccharides), Phenylalanine, C–CI stretching, C–C skeletal, C–C stretching, Amide III, Beta-lactamase enzyme, Amide III, Peptidoglycan (glucosamines) from the cell wall, CH deformation, Guanine and adenine ring stretching and Amide I, respectively. Substances showing changes upon plantaricin treatment are believed to be components of the cell membrane or cell wall. Specifically, the increase in the intensity of the 645 cm^-1^ peak indicates changes of bacterial proteins [22], while shifts at 1380 cm^-1^ suggest alterations in cell wall strength and stability [28]. The reduction in intensity for peaks of bacteriocin-treated samples is likely to be caused by damage to the cell membrane or cell wall, rendering these substances undetectable. Among the five plantaricin combinations tested, spPlnE&J and spPlnE&F groups showed the most significant peak reductions, suggesting that these bacteriocins exhibited the highest efficacy.

To visually assess effects of plantaricin treatment, PCA was performed using Raman peaks obtained in the 300–1900 cm^-1^ range (Fig. 1B). The untreated control group was located in the fourth quadrant, whereas samples treated with plantaricins, with the exception of spPlnA, were distributed in the first, second, and third quadrants. Notably, spPlnE&J and spPlnE&F groups, which exhibited the most significant changes in Raman peaks, were located in the first and second quadrants, showing the most distinct pattern compared to the control group.

To validate PCA results, PLS-DA was conducted. PLS-DA results were consistent with those of the PCA (Fig. 1C). The agreement between these two analytical methods is considered reliable [30]. In PLS-DA results, the untreated control group clustered in the third quadrant, whereas plantaricins-treated samples were spread across other quadrants. Consistent with PCA results, spPlnE&J and spPlnE&F groups were located in the first and second quadrants, clearly separated from the control group. These results indicated that plantaricin combinations of spPlnE&J and spPlnE&F had a stronger impact on *L. monocytogenes*. These findings confirm that synthetic plantaricins can induce significant changes in molecular compositions of *L. monocytogenes*, supporting their potential as effective antimicrobial agents. However, although these synthetic plantaricins exhibited distinct antibacterial activities, further studies are required to fully distinguish the specific effects of different treatments

### Synthetic Antimicrobial Substances Cause Cell Lysis of *L. monocytogenes* ATCC 19111

**Fig. 2:**
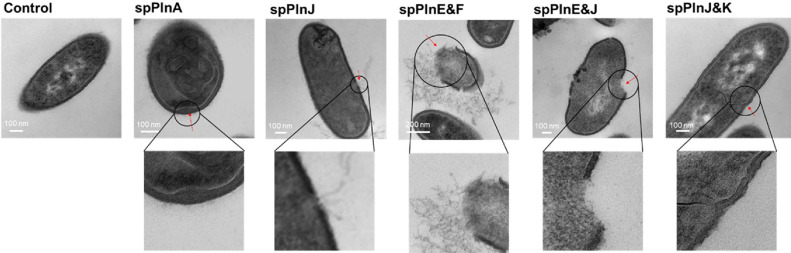
TEM for *L. monocytogenes* ATCC 19111 treated with synthetic plantaricins. Bacterial ultrastructure was examined using JEM−1010 transmission electron microscopy. These bacteria were severely affected, particularly by synthetic plantaricins. Debris from decomposition of the cell wall is marked by red arrow.

Raman spectroscopy findings indicated potential disruptions to the cell wall structure of *L. monocytogenes* ATCC 19111 when treated with synthetic plantaricins. To further validate these observations, we employed TEM to directly visualize structural changes. TEM analysis was conducted for samples treated with five synthetic plantaricins (spPlnA, spPlnJ, spPlnE&F, spPlnE&J, and spPlnJ&K) to characterize the resulting damage to *L. monocytogenes* ATCC 19111. Treated samples displayed notable signs of cell wall disruption, with evidence of lysis and fragmentation around the cell envelope (Fig. 2).

Among the five plantaricin samples, treatments with spPlnE&F and spPlnE&J showed cell membrane and cell wall destruction in *L. monocytogenes* ATCC 19111, resulting in cytoplasmic leakage. Although cell lysis was less extensive with spPlnJ treatment than with spPlnE&F and spPlnE&J, cytoplasmic leakage was still observed. For spPlnA and spPlnJ&K, which showed the lowest MIC, cell lysis was not detected, although separation of the cell membrane from the cell wall was observed. Structural alterations observed through TEM aligned closely with results obtained from Raman spectroscopy, reinforcing the notion that synthetic plantaricins, especially spPlnE&F and spPlnE&J, could effectively destabilize the cell wall of *L. monocytogenes* ATCC 19111, thus elucidating their mode of antimicrobial action. In contrast, spPlnA and spPlnJ&K seemed to inhibit growth of *L. monocytogenes* ATCC 19111 through a mechanism other than cell lysis.

### Stability of Heat, pH, and Enzymes on Antibacterial Activity

Synthetic plantaricins were tested for their antibacterial activities *against* *L. monocytogenes* under varying temperature, pH, and enzyme conditions. Results showed that all tested combinations except spPlnJ retained their antibacterial potency when exposed to temperatures below 40°C (Table 1). In the case of spPlnJ, its activity decreased at 30°C and completely lost at 40°C. Additionally, the antibacterial activity of combinations was preserved across a pH range from 4 to 7 (Table 1). Since this pH range aligns with typical conditions found in many food products, these bacteriocin combinations have potential for broad applications in the food industry. Regarding pH stability, all plantaricin combinations retained their antibacterial effectiveness across a pH range of 4 to 7, suggesting suitability for applications in various food matrices where pH can vary (Table 1). This stability across different pH conditions suggests that these bacteriocins could be applied in diverse food processing environments without losing their effectiveness.

Plantaricin is a peptide-based antimicrobial substance. It lost its antibacterial activity when exposed to protease K, an enzyme that breaks down proteins by cleaving peptide bonds (Table 1). However, plantaricin combinations—spPlnA, spPlnJ, spPlnE&F, and spPlnE&J—did not lose their activities when exposed to α-amylase and lysozyme except for spPlnJ&K. These results indicate that synthetic plantaricins hold promise as natural preservatives in non-sterilized environments such as food fermentation processes.

## Discussion

This study demonstrates that synthetic plantaricins derived from *Lb. plantarum* KM2 exhibits antimicrobial activities against *L. monocytogenes*, a major foodborne pathogen (Figs. 1 and 2). Specifically, TEM results suggest that spPlnE&F and spPlnE&J can inhibit *L. monocytogenes* by disrupting its cell wall (Fig. 2). However, spPlnA and spPlnJ&K appear to inhibit the growth of *L. monocytogenes* through protoplast formation rather than cell wall degradation (Fig. 2). This study demonstrates that synthetic plantaricins derived from *Lb. plantarum* KM2 exhibit antimicrobial activities against *L. monocytogenes*, a major foodborne pathogen (Figs. 1 and 2). Specifically, TEM results suggest that spPlnE&F and spPlnE&J can inhibit *L. monocytogenes* by disrupting its cell wall (Fig. 2). However, spPlnA and spPlnJ&K appear to inhibit the growth of *L. monocytogenes* through protoplast formation rather than cell wall degradation (Fig. 2). Two-peptide bacteriocins are known to form pores that cause cell lysis through interaction via the GxxxG motif of alpha-peptides, which contain positively charged amino acids for inner-membrane binding, and beta-peptides, which bind to the outer membrane [31, 32]. While previous studies have attempted to categorize plantaricin E, F, J, and K into alpha- and beta-peptides, a clear distinction has not been established yet. Nevertheless, the observed efficacy of spPlnE and spPlnJ in this study implies the possibility of their interaction. Further research is recommended to investigate their binding mechanisms on the membrane.

In this study, instead of assessing the antimicrobial activity of plantaricin produced by KM2, peptides synthesized based on putative plantaricin amino acid sequences derived from KM2's genome were used. Their antimicrobial activities were tested using various combinations. Antimicrobial activity was evaluated through Raman spectroscopy and TEM. Raman spectroscopy is particularly effective for detecting changes on microbial surfaces by analyzing interactions of photons with microbes [33]. Bacteriocins typically act on microbial cell walls or membranes to kill or inhibit the growth of target microorganisms [34]. Therefore, employing Raman spectroscopy to detect surface changes in microbes is an efficient approach. In this study, Raman spectroscopy was utilized to assess changes in microbial surfaces. As freeze-dried samples were used, sufficient signals could be obtained without extensive preprocessing, thus saving time and costs while allowing for a simpler and more efficient analysis. When plantaricins were applied, changes in the peaks were observed, with variations in peak increases and decreases depending on the species. For example, when synthetic plantaricin was applied to *Flavobacterium* sp. KCCM 11374, the Raman spectrum showed increased peaks compared to the untreated control [19]. Conversely, for *L. monocytogenes*, peaks decreased relative to the control (Fig. 1A). The target strain *Flavobacterium* sp. is a Gram-negative bacterium, while *L. monocytogenes* is a Gram-positive bacterium. Differences in peak patterns might have resulted from structural differences in their cell walls. In previous studies, when a Gram-negative strain of *E. coli* KACC 10005 was treated, peaks increased compared to the untreated control [35]. However, treating *E. coli* DH5α with ampicillin resulted in decreased peaks compared to the control [36]. While peak differences were observed between treated and untreated control groups when antimicrobial agents were applied, these differences did not consistently correlate with the Gram-positive or Gram-negative nature of bacteria. Moreover, peak increases or decreases did not show a uniform trend [37-39]. In conclusion, Raman peak changes in the presence of antimicrobial agents are different compared to control groups, rather than showing a consistent trend of increase or decrease. Despite this, observed peak changes demonstrate that Raman spectroscopy is a valuable tool for evaluating effects of antimicrobial agents.

To date, several plantaricins derived from *Lb. plantarum*, including Plantaricin Q7, Plantaricin UG1, and Plantaricin NA, have been reported to exhibit antimicrobial activity against *L. monocytogenes* [40-42]. Consequently, these findings suggest that plantaricins produced by *Lb. plantarum* may serve as effective bacteriocins against *L. monocytogenes*. However, comparative analyses of the amino acid sequences of these plantaricins, their correlations with antimicrobial efficacy, and investigations into their precise regulatory pathways and mechanisms of action remain limited. Therefore, comprehensive studies addressing these aspects are warranted.

Finally, although the supernatant of the KM2 strain did not exhibit antimicrobial activity against *L. monocytogenes*, synthetic plantaricin peptides derived from KM2's genetic profile demonstrated strong antimicrobial effects, particularly for specific combinations. This discrepancy is likely due to a low concentration of plantaricin in the supernatant. Further qualitative and quantitative analyses of plantaricin produced by the KM2 strain are necessary to confirm this.

In conclusion, the antimicrobial effect of synthetic plantaricin against *L. monocytogenes* was found to occur through mechanisms such as cell lysis (*e.g.*, spPlnJ, spPlnE&F, and spPlnE&J) or changes to the cell wall (*e.g.*, spPlnA and spPlnJ&K) (Fig. 2). These synthetic plantaricins could be utilized to inhibit growth of *L. monocytogenes*, potentially contributing to the reduction of food spoilage or foodborne illnesses. Additionally, applying KM2-derived plantaricin-producing strains as starter cultures in fermented foods could serve as an effective strategy to reduce spoilage and foodborne illnesses in these products.

## Figures and Tables

**Table 1:** Antibacterial activities of synthetic plantaricins with various combinations against *L. monocytogenes* ATCC 19111.

	spPlnA	spPlnJ	spPlnE&F	spPlnE&J	spPlnJ&K
Stability of temperature					
20°C	1.4 ± 0.1^a^	1.2 ± 0.1^b^	1.7 ± 0.1^c^	1.6 ± 0.1^d^	1.3 ± 0.1^b^
30°C	1.4 ± 0.0^a^	0.5 ± 0.1^b^	1.7 ± 0.1^c^	1.5 ± 0.0^d^	1.2 ± 0.1^e^
40°C	1.1 ± 0.1^a^	0.0 ± 0.0^b^	1.5 ± 0.1^c^	1.4 ± 0.1^c^	1.2 ± 0.1^a^
pH stability					
pH4	1.2 ± 0.0^a^	1.1 ± 0.1^ab^	1.5 ± 0.1^c^	1.5 ± 0.0^c^	1.1 ± 0.0^b^
pH5	1.3 ± 0.1^a^	1.1 ± 0.1^a^	1.5 ± 0.2^b^	1.5 ± 0.1^b^	1.1 ± 0.1^a^
pH6	1.5 ± 0.1^a^	1.1 ± 0.0^b^	1.6 ± 0.1^c^	1.5 ± 0.1^a^	1.2 ± 0.1^d^
pH7	1.4 ± 0.1^a^	1.1 ± 0.0^b^	1.7 ± 0.1^c^	1.4 ± 0.2^ad^	1.2 ± 0.1^bd^
Stability of enzyme					
α-Amylase	1.4 ± 0.1^a^	1.1 ± 0.1^b^	1.4 ± 0.1^a^	1.3 ± 0.1^a^	0.7 ± 0.1^c^
Proteinase K	–	–	–	–	–
Lysozyme	1.4 ± 0.1^a^	1.1 ± 0.1^b^	1.5 ± 1.1^a^	1.4 ± 0.1^a^	0.5 ± 0.1^c^

Different superscripts in a row indicate significant differences at *p* < 0.05 according to Duncan’s multiple-range test. “–” means that a clear zone was not formed.
